# The effects of microbiota abundance on symptom severity in Parkinson’s disease: A systematic review

**DOI:** 10.3389/fnagi.2022.1020172

**Published:** 2022-12-08

**Authors:** Eliša Papić, Valentino Rački, Mario Hero, Zoran Tomić, Nada Starčević-Čižmarević, Anja Kovanda, Miljenko Kapović, Goran Hauser, Borut Peterlin, Vladimira Vuletić

**Affiliations:** ^1^Department of Neurology, Faculty of Medicine, University of Rijeka, Rijeka, Croatia; ^2^Clinic of Neurology, Clinical Hospital Center Rijeka, Rijeka, Croatia; ^3^Department of Medical Genetics and Biology, Faculty of Medicine, University of Rijeka, Rijeka, Croatia; ^4^Clinical Institute of Genomic Medicine, Ljubljana University Medical Center, Ljubljana, Slovenia; ^5^Department of Internal Medicine, Faculty of Medicine, University of Rijeka, Rijeka, Croatia

**Keywords:** microbiota, Parkinson’s disease, motor symptoms, systematic review, relative abundance

## Abstract

**Introduction:**

Parkinson’s disease (PD) is neurodegenerative disease with a multifactorial etiopathogenesis with accumulating evidence identifying microbiota as a potential factor in the earliest, prodromal phases of the disease. Previous research has already shown a significant difference between gut microbiota composition in PD patients as opposed to healthy controls, with a growing number of studies correlating gut microbiota changes with the clinical presentation of the disease in later stages, through various motor and non-motor symptoms. Our aim in this systematic review is to compose and assess current knowledge in the field and determine if the findings could influence future clinical practice as well as therapy in PD.

**Methods:**

We have conducted a systematic review according to PRISMA guidelines through MEDLINE and Embase databases, with studies being selected for inclusion via a set inclusion and exclusion criteria.

**Results:**

20 studies were included in this systematic review according to the selected inclusion and exclusion criteria. The search yielded 18 case control studies, 1 case study, and 1 prospective case study with no controls. The total number of PD patients encompassed in the studies cited in this review is 1,511.

**Conclusion:**

The link between gut microbiota and neurodegeneration is a complex one and it depends on various factors. The relative abundance of various microbiota taxa in the gut has been consistently shown to have a correlation with motor and non-motor symptom severity. The answer could lie in the products of gut microbiota metabolism which have also been linked to PD. Further research is thus warranted in the field, with a focus on the metabolic function of gut microbiota in relation to motor and non-motor symptoms.

## Introduction

Parkinson’s disease (PD) is the second most common neurodegenerative disease in the world (Van Den Eeden et al., 2003). It mainly presents with a triad of symptoms including rigor, bradykinesia, and tremor (Kouli et al., 2018), along with a variety of other motor and non-motor symptoms (Poewe et al., 2017).

It is postulated that the main pathophysiological mechanism lies in the accumulation of α–synuclein in the brain, primarily in the substantia nigra, which leads to the loss of dopaminergic neurons and the typical symptoms of PD. As the disease progresses, said changes spread to other regions of the brain, causing neurodegeneration, slowly leading to severe motor and cognitive impairment (Shulman et al., 2011). PD is a multifactorial condition, with causes ranging from exposure to various environmental factors, such as pesticides (Chen and Ritz, 2018), traumatic brain injury (Delic et al., 2020), gene mutations (Klein and Westenberger, 2012), and more recently, microbiota (Fitzgerald et al., 2019).

Novel research has identified microbiota as a potential factor in the earliest prodromal phases of the disease (Shen et al., 2021). The mechanism behind this is thought to lie in the gut-brain axis, a complex bidirectional system of communication between the intestines and the brain (Carabotti et al., 2015), with various potential pathways described with the vagal nerve (Breit et al., 2018) and the proven transneuronal propagation of α–synuclein from the gut to the brain being the most promising in the research of gut microbiota influence on the brain (Kim et al., 2019). It has been shown in previous studies that the underlying changes in the gut that could potentially lead to this pathological retro-axonal transport include microbiota composition, with the composition greatly differing in PD patients when compared to the healthy controls (Pereira et al., 2017), as well as microbiota metabolic function, mainly through the secretion of various SCFA (Shen et al., 2021). Alterations in the microbiome could potentially lead to prodromal symptoms such as hyposmia and GI dysfunction (Pereira et al., 2017), as well as modulating motor symptoms in the later stages of the disease (Sampson et al., 2016).

The changes in the gut microbiome have also been linked with the response to PD therapy, especially levodopa (Keshavarzian et al., 2020). It is thus clear that the potential effects of dysbiosis could play a part in the prodromal, but also in the latter stages of the disease and as such should be carefully studied further (Fitzgerald et al., 2019). Studies have already shown that there are certain potential therapeutic approaches that could be taken to prevent or reverse the changes in the microbiome and consequently modulate the disease course and severity. For example, potential beneficial effects have been proposed in the application of antibiotics (Li et al., 2004; González-Lizárraga et al., 2017; Pu et al., 2019), probiotics (Cassani et al., 2011; Surwase and Jadhav, 2011; Srivastav et al., 2019), prebiotics (Cantu-Jungles et al., 2019; Qiao et al., 2020), dietary intervention (Watson et al., 2018; Hegelmaier et al., 2020), and fecal microbiota transplant (FMT) (Xue et al., 2020). The field of research is still growing, and further studies could reveal additional therapeutic approaches targeting the microbiome.

Our aim in this systematic review is to compose and assess current knowledge in the field and determine if the findings could influence future clinical practice as well as therapy in PD.

## Methods

### Search strategy

**FIGURE 1:**
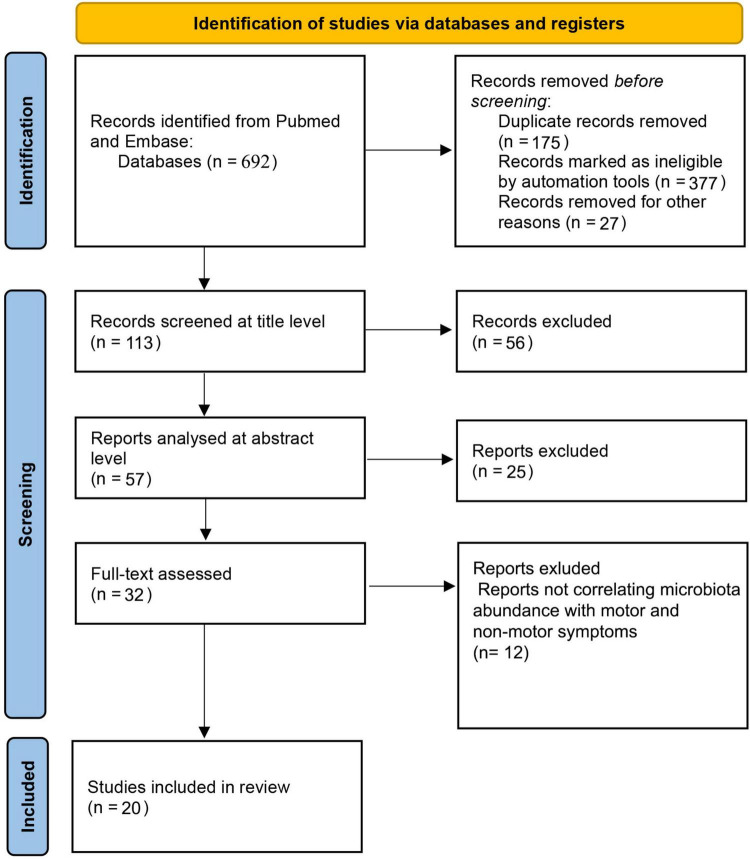
PRISMA flow diagram.

We have conducted a systematic review according to PRISMA guidelines (Page et al., 2021). Our search was focused on the MEDLINE and Embase databases. The search was done on articles published from January 1st of 2012 up to June 1st of 2022. We used the following keywords on all fields and MeSH terms: “PD,” “microbiota,” “microbiome,” along with Boolean terms “AND” and “OR.” The search rendered 692 records after we applied appropriate filters. The studies were then selected based upon the following inclusion and exclusion criteria (Figure 1). Articles were first screened by title and abstract, followed by full-text checking for their eligibility. The selection of articles was done independently by 5 authors (EP, VR, MH, ZT, AK), and final inclusion was done by agreement.

### Inclusion and exclusion criteria

Studies accepted for inclusion were: (a) studies with patients diagnosed with PD; (b) studies published from January 1st of 2012 up to June 1st of 2022; (c) studies published in the English language; (d) studies published in indexed and peer-reviewed journals; (e) studies that evaluated motor symptoms through the Unified PD Rating Scale Part III (UPDRS III) and/or Hoehn and Yahr disease stage progression in correlation with gut microbiota abundance (f) studies that evaluated non-motor symptoms through the Unified PD Rating Scale Part I (UPDRS I), Non-Motor Symptoms Scale (NMSS), Non-Motor Symptoms Questionnaire (NMSQ), Mini Mental State Examination (MMSE), and Montreal Cognitive Assessment (MoCA) in correlation with gut microbiota abundance.

Exclusion criteria include: (a) studies published in regional languages other than English, (b) studies not correlating gut microbiota abundance with motor and/or non-motor symptoms through verified clinical scales. Study design was evaluated for type, and 20 studies were finally included in the review (Table 1).

**TABLE 1:** Cited studies listed by study design.

References	Study design	Population	Composition and correlation
Scheperjans et al. (2015)	Case-control study	72 patients and 72 controls	Family *Prevotellaceae*: relative abundance negatively correlates with UPDRS III
Aho et al. (2019)	Case-control study	64 PD patients, controls	Family *Prevotellaceae* less abundant in progressed PD (H&Y) Genus *Prevotella* more abundant in stable subjects
Lin et al. (2019)	Case-control study	80 PD patients and 77 controls	Genus *Bacteroides:* relative abundance positively correlated with motor symptom severity
Heintz-Buschart et al. (2018)	Case—control study	76 PD patients, 21 idiopathic REM sleep behavior disorder patients, and 7 controls	Genus *Akkermansia* more abundant in PD patients—related to non-motor symptoms. The relative abundances of *Anaerotruncus* spp., *Clostridium XIVa*, and *Lachnospiraceae* family and genus *Akkermansia* positively correlated with UPDRS III *Anaerotruncus* species related to depression in Parkinson’s disease
Li et al. (2022)	Case-control study	91 PD patients and 91 healthy controls (HC)	*Clostridia, Clostridiales, and Ruminococcaceae* negatively correlated with MMSE
Barichella et al. (2019)	Prospective observational case-control study	350 patients (193 PD, PSP 22, MSA 22; HC 113)	Higher abundance of *Christensenellaceae* linked with worse non-motor symptoms *Lactobacillaceae* positive correlation with UPDRS III. *Lachnospiraceae* negative correlation with UPDRS III.
Zhang et al. (2020)	Case-control study	63 PD patients, 63 healthy spouses (HS) and 74 healthy people (HP)	*Parabacteroides, Akkermansia, Coprococcus, Bilophila, Collinsella, Methano-brevibacter, Eggerthella, Adlercreutzia* associated with PD progression and symptom severity.
Li et al. (2019)	Case-control study	51 PD patients and 48 healthy controls	Relative abundances of families *Acidaminococcaceae, Erysipelotrichaceae*, genera *Phascolarctobacterium, Coprococcus, Tyzzerella*, and species *Ruminococcus_torques* showed a positive correlation with UPDRS III Relative abundances of families *Acidaminococcaceae, Erysipelotrichaceae*, genera *Phascolarctobacterium, Akkermansia, Coprococcus, Tyzzerella*, and species *Ruminococcus_torques* showed a positive correlation with non-motor symptoms (NMSQ and SCOPA) Relative abundances of order *Bacillales* and species *Pseudomonas_veronii* showed a negative correlation with UPDRS III Relative abundance of order *Lactobacillales* is negatively correlated with NMSQ
Murros et al. (2021)	Case-control study	20 PD patients and 20 healthy controls.	Eleven patients with a more severe disability of PD had a significantly higher amount of *DSV* bacteria than the nine patients that were classified below 2.0 points under the Hoehn-Yahr system; *DSV* bacteria were significantly more abundant in patients with hyposmia
Rosario et al. (2021)	Case-control study	26 drug naive PD and 25 controls, [11 healthy controls (COs), 14 diseased controls (DCs) with cardiovascular risk factors]	The abundance of the genus *Erysipelatoclostridium*, the species *E. coli* and the species *Victivallis vadensis* was positively correlated with UPDRS III and age in people with PD + *E. coli* and *V. vadensis* also linked to GI dysfunction
Li et al. (2017)	Case-control study	24 PD patients; 14 controls	*Genera:* The relative abundance of *Faecalibacterium* decrease in severe Parkinson compared to mild Parkinson and control (H&Y and UPDRS III) *Megasphaera* relative abundance increased in severe Parkinson compared to mild Parkinson (H&Y and UPDRS III) and control UPDRS III scores positively correlated with *Enterococcus, Proteus*, and *Escherichia-Shigella* UPDRS III negatively correlated with *Blautia Faecalibacterium, Ruminococcus, Haemophilus*, and *Odoribacter*
Qian et al. (2018)	Case-control study	45 PD patients, 45 healthy controls	*Butyricicoccus* and *Clostridium XlVb* positively associated with MMSE scores
Pietrucci et al. (2019)	Case-control study	80 PD and 72 controls	Lower levels of *Lachnospiraceae* and higher levels of *Enterobacteriaceae* families correlated with increased disease severity and motor impairment
Takahashi et al. (2022)	Case study	223 PD patients	*Blautia* significantly decreased and *Lactobacillus* significantly increased in PD patients with motor complications
Mertsalmi et al. (2017)	Case-control study	74 PD patients with 75 controls	Lower abundance of the family *Prevotellaceae* as well as the genera *Prevotella* and *Bacteroides* in IBS like symptoms in PD patients
Baldini et al. (2020)	Case-control study	147 typical PD cases, 162 controls	Relative abundances of genera *Peptococcus* and *Flavonifractor* are positively correlated with UPDRS III Relative abundance of genus *Bilophila* is positively correlated with Hoehn and Yahr Relative abundance of genus *Paraprevotella* is negatively correlated with UPDRS III and Hoehn and Yahr Genus *Bifidobacterium* is positively correlated with constipation
Minato et al. (2017)	Prospective study	36 PD patients divided into deteriorated and stable groups	Low count of *Bifidobacterium* at year 0 was associated with worsening of UPDRS I (hallucinations) no individual bacterial groups/genera/species at year 0 were correlated with worsening of total UPDRS scores in 2 years
Ren et al. (2020)	Case-control study	13 PD patients PD-MCI/14 PD –NC (normal cognition) and 13 healthy controls	Relative abundances of genera *Ruminococcus, Bilophila, Desulfovibrio, Barnesiella, Butyricimonas, Acidaminococcus, Pyramidobacter*, and *Oxalobacter* were negatively associated with the MMSE scores; Relative abundances of genera *Alistipes, Sutterella, Odoribacter, Butyricimonas, Hungatella, Helicobacter, Solobacterium, Oscillospira*, and *Hydrogenoanaerobacterium* were negatively associated with the MoCA scores
Cosma-Grigorov et al. (2020)	Case-control multivariate study	71 PD patients and 30 healthy	Significant positive correlation of *Parabacteroides* and *Turicibacter* with disease duration and UPDRS III
Weis et al. (2019)	Case-control study	34 PD and 25 controls	H and Y 1–2.5: significantly increased relative abundance of *Peptoniphilus* and F*aecalibacterium* compared to controls H and Y 3–4: significant increase in the relative abundance of *Peptoniphilus*

PD, Parkinson’s disease; HC, healthy controls; PSP, progressive supranuclear palsy; MSA, multiple system atrophy.

### Clinical scales for the evaluation of motor and non-motor symptoms in Parkinson’s disease used in the selected studies

Unified PD Rating Scale is a widely used clinical scale for the evaluation of both motor and non-motor symptoms in PD and it is split into four parts. Part I concerns non- motor experiences of daily living and covers areas such as sleep and mood, Part II covers motor experiences of daily living such as hygiene, clothing and other daily activities requiring healthy motor skills, Part III measures the severity for motor symptoms such as rigor, tremor, bradykinesia, and others, while the Part IV is used to describe eventual motor complications (i.e., dyskinesia) (Goetz et al., 2008). In the studies collected for this systematic review, UPDRS III was used in correlation between motor symptom severity and microbiota relative abundance, while UPDRS I was used in some studies to express non-motor symptom severity.

The Hoehn and Yahr (H&Y) system is used to grade the severity of PD symptoms and is expressed as a scale of 1–5. The stages 1–3 represent patients who are considered to be minimally disabled, while stages 4 and 5 represent patients who are considered to be severely disabled. Besides representing the motor symptom severity linked with disease progression, the H&Y scale has also been positively correlated with cognitive decline and dementia in PD patients (Modestino et al., 2018).

The NMSQ is a patient-based screening tool for the presence of non-motor symptoms ranging from hyposmia, incontinence, sexual performance to hallucinations and diplopia. It does not evaluate the severity of the symptoms (Chaudhuri et al., 2006). On the other hand the NMSS is used for evaluation of the severity of non-motor symptoms and uses a 30-item rater-based scale to cover a wide array of non-motor symptoms, rating their severity on the scale of 0–3 as well as their frequency on the scale of 1–4 (Chaudhuri et al., 2007).

The MoCA is a screening tool used for the evaluation of cognitive impairment and covers various cognitive domains such as visuospatial abilities, executive functions, short-term memory recall, language, abstract reasoning, orientation and more. It contains 30 points, with a score of 26 or over is considered to be normal (Nasreddine et al., 2005).

Similarly to MoCA, the Mini-Mental State Examination (MMSE) is also a 30-point questionnaire used for the measurement of cognitive impairment. It requires less time for administration, and can help differentiate between different types of dementia such as Alzheimer’s disease or PD dementia (Pangman et al., 2000). It should be noted that both MoCA and MMSE are used mostly as screening tools, and are by no means specific enough without follow-up imaging and additional diagnostic procedures to provide a final diagnosis.

### Methods for microbiota analysis implemented in the selected papers

The papers analyzed in this reviewed have used a wide array of different methods for microbiota isolation and sequencing, taxonomic assignment and clustering through Operational Taxonomic Units (OTU) and compositional and statistical analysis. The information has been included in Table 2 and further covered and discussed in the Discussion section.

**TABLE 2:** Methods for microbiota isolation, sequencing, taxonomic assignment, data, and statistical analysis.

References	Methods for microbiota isolation and sequencing	Taxonomic assignment of operational taxonomic unit (OTU)	Microbiota data analysis methods and statistical methods
Scheperjans et al. (2015)	Stool samples. Pyrosequencing of V1–V3 regions of 16SRNA	Mothur’s Standard Operating Procedure (SOP) for MiSeq	*t*-test, Mann-Whitney *U*-test, Fisher’s exact test, Metastats. Generalized linear model (GLM) for the distribution of bacterial abundances, Spearman correlation coefficient for correlations between rel. abundances and clinical factors; other methods
Aho et al. (2019)	Stool samples. V3–V4 regions of 16S RNA PCR amplification.	Mothur’s Standard Operating Procedure (SOP) for MiSeq	*t*-test, Wilcoxon signed rank test, Fisher’s exact test, False discovery rate (FDR), phyloseq. ANCOM, DESeq2, random forests for the distribution of abundances; Spearman correlation coefficient; other methods
Lin et al. (2019)	Stool samples. V4 16s RNA PCR amplification	Quantitative Insights Into Microbial Ecology (QIIME) 1.9.1; classification based on the Greengenes gg_13_8 database (Miseq)	*t*-test, chi-square test, ANOVA, Levene’s test, Mann-Whitney *U*-test, Kruskal-Wallis test; ANCOM, Linear discriminant analysis (LDA) effect size (LEFSe), FDR; other methods
Heintz-Buschart et al. (2018)	Stool samples. V4 region of 16S RNA and 18S RNA PCR amplification. Shotgun sequencing	LotuS R (R Foundation for Statistical Computing, Vienna, Austria) HiSeq	Permutational multivariate analysis of variance, Fisher’s exact test, Kruskal-Wallis test, phyloseq, Mann-Whitney *U*-test, DESeq2, ANCOM, FDR; other methods
Li et al. (2022)	Stool samples. V4–V5 regions of 16S RNA amplification and sequencing	Uparse software with Mothur algorithm, MUSCLE software	*t*-test, Wilcoxon’s rank-sum test, (LDA) effect size (LEFSe), random forests, Spearman correlation coefficient, GLM for elimination of confounding factors; other methods
Barichella et al. (2019)	Stool samples. V3–V4 regions of 16S RNA amplification and sequencing	QIIME pipeline; data clustered and taxonomically assigned via Ribosomal Database Project (RDP) classifier against a Greengenes database. MiSeq	R package “vegan,” multivariate GLM (negative binomial distribution with log link), regression analysis, P-MANOVA, Spearman correlation coefficient and others
Zhang et al. (2020)	Stool samples. V4 region of 16S RNA amplification and sequencing	QIIME2 pipeline, DADA2. Clustering via VSEARCH against Greengenes 13_7 HiSeq	R, Fisher’s exact test, Kruskal-Wallis, LDA effect size (LEfSe), Spearman correlation coefficient, and others
Li et al. (2019)	Stool samples. V4 region of 16S RNA amplification and sequencing	Uparse v7.0.1001. Mothur. SILVA SSU rRNA database.	R, *t*-test, Wilcoxon rank-sum test, Linear discriminant analysis. (LDA) effect size (LEfSe) analysis, FDR, Spearman correlation coefficient, and others
Murros et al. (2021)	Stool samples. Primers for 16S rRNA—for specific detection of *Desulfovibrio* genus and subspecies. PCR amplification.	Not applicable	Multiple statistical tests (Fisher’s exact test; strength of association tested by Phi and Cramer’s *V*-test; Mann Whitney *U*-test for comparison of DSV in PD vs. controls and patients with high vs. low levels of disease progression) and others
Rosario et al. (2021)	Shotgun metagenomic data from a German PD Cohort	DirichletMultinomial	Wilcoxon signed-rank test, FDR, Spearman correlation coefficient, Cytoscape (integrative correlation network), and others
Li et al. (2017)	Stool samples. V3–V5 region of 16S RNA amplification and sequencing	Mothur and USEARCH (v8.0) SILVA 16S rRNA database v119. Mothur SOP for MiSeq.	Metastats method for abundance features; R, UniFrac distance metrics analysis, Spearmen’s rank correlation. Kruskal-Wallis-test, t-test, Chi-squared test)
Qian et al. (2018)	Stool samples. V3–V4 region of 16S RNA amplification and sequencing	QIIME OTU assigned using UPARSE. Reference database - Ribosomal Database Project (RDP).	R software, statistical tests (*t*-test, Pearson’s Chi-square test), GLM, RF, Spearmen’s correlation analysis, LASSO (least absolute shrinkage and selection operator), and others
Pietrucci et al. (2019)	Stool samples. V3–V4 regions of 16S RNA amplification and sequencing	QIIME 1.9.1. USEARCH 6.1 and GreenGenes 13.8	GLM, Willcoxon-Mann-Whitney. DESeq2, PERMANOVA test, regression analysis, and others
Takahashi et al. (2022)	Stool samples. V3–V4 regions of 16S RNA amplification and sequencing	QIIME2 DADA2 SILVA taxonomy database release 132 (60)	Wilcoxon rank-sum test, ANCOVA, GLM, Bonferroni correction, and others
Mertsalmi et al. (2017)	Stool samples. V1–V3 region of 16S RNA—amplification and pyrosequencing	Mothur’s Standard Operating Procedure (SOP) for MiSeq	*T*-test; Mann—Whitney test; Fisher’s two sided exact test. Microbiome data: Phyloseq, DESeq2 package 14 (based on binomial generalized linear models); FDR
Baldini et al. (2020)	Stool samples. V3–V4 regions of 16S RNA amplification and sequencing	SPINGO (SPecies level IdentificatioN of metaGenOmic amplicons) classifier	Genome scale metabolic reconstructions; flux balance analysis (FBA), community metabolic modeling; fractional regression (family of GLM), FDR correction
Minato et al. (2017)	Stool samples. PCR of 16S or 23S RNA	Composition of gut microbiota was analyzed using the Yakult intestinal Flora-SCAN (YIF-SCAN), which exploited qRT-PCR of bacterial 16S or 23S rRNA using SYBR Green I. 19 bacterial taxa were preselected based on high prevalence in the human intestines, frequently observed pathogens, and preference of the Yakult company that merchandises *Lactocbacillus*-containing yoghurt. Other data was not included.	Wilcoxon signed ranked test; Fisher’s exact test; Pearson correlation; FDR
Ren et al. (2020)	Stool samples. V3–V4 regions of 16S RNA amplification and sequencing. Gas Chromatography and Mass Spectrometry (GC-MS)	Mothur, UPARSE and R. UPARSE pipeline used for OTU clustering. Silva 128 database used for assignment of representative OTU sequences.	Shapiro-Wilk test; Pearson chi-square; Bonferroni adjustment; *t*-test; LDA effect size (LEfSe), GLM, DESeq; predictions via KEGG orthologs; Kruskal-Wallis test
Cosma-Grigorov et al. (2020)	Stool sample. V3–V4 regions of 16S RNA amplification and sequencing	Usearch and greengenes 16S rRNA database v13.5	Kolmogorov-Smirnov test, *t*-test, Mann-Whitney *U*-test, Fisher’s exact test, *z*-test with Bonferroni correction; abundance—Kruskal Wallis, Wilcoxon signed test. Sparce correlation (SparCC) in MicrobiomeAnalyst and others
Weis et al. (2019)	Stool sample. V4–V5 region of 16S RNA amplification and sequencing	QIIME 1.9.1. SILVA database for taxonomy assignment	ANOVA, Wilcoxon-Mann-Whitney test; FDR, Spearman correlation analysis, and others

## Results

The primary search yielded a total of 692 studies using the described method and search parameters. 113 studies remained after excluding duplicate records and filtering them out with automation tools. These were screened on the title level and 56 studies were excluded, leaving 57 studies that were analyzed on the abstract level, where additional 25 studies were excluded. The full text was analyzed for 32 studies, and additional 12 studies were excluded (not correlating microbiota abundance with motor and/or non-motor symptoms; *n* = 12). Therefore, 20 studies were included in this systematic review according to the selected criteria. The complete PRISM flow chart for this systematic review is given in Figure 1. When looking at study designs, the search yielded 18 case control studies, 1 case study, and 1 prospective case study with no controls. The total number of PD patients encompassed in the studies cited in this review is 1,511.

### Microbiota abundance in relation to motor and non-motor symptoms

The link between the composition of the gut microbiota and PD symptoms has been scarcely researched so far. Most of the studies included in this systematic review analyzed the abundance of gut microbiota from the feces through amplification and sequencing methods of the different regions of the bacterial 16s ribosomal gene. They were then correlated with either UPDRS and the modified Hoehn and Yahr scale or through non-motor symptom scales and questionnaires such as NMSS and NMSQ, as well as through cognitive tests, more specifically MoCA and MMSE.

#### Impact of microbiota on motor symptoms in Parkinson’s disease

Most papers analyzed in this systematic review suggest a positive correlation of gut microbiota abundance with motor symptoms and disease severity in PD (Table 3).

**TABLE 3:** Relative abundances of bacterial taxa in correlation with motor symptoms.

Kingdom	Phylum	Order	Family	Genus
*Bacteria*	*Bacillota*—positive correlation with UPDRS III	*Eubacteriales*	*Oscillospiraceae*	*Ruminococcus—*negative correlation with UPDRS III
				Mediterraneibacter* species *Ruminococcus_ torques*—positive correlation with UPDRS III
				*Anaerotruncus*—positive correlation with UPDRS III
				*Faecalibacterium*—negative correlation with UPDRS III/H&Y
			*Lachnospiraceae—*negative correlation with UPDRS III	*Coprococcus*—positive correlation with UPDRS III
				*Tyzzerella*—positive correlation with UPDRS III
				*Blautia*—negative correlation with motor complications
				*Clostridium cluster XIVa*—positive correlation with UPDRS III
			*Peptoniphillaceae*	*Peptoniphilus*—positive correlation with H&Y
		*Clostridiales*	*Peptococcaceae*	*Peptococcus*—positive correlation with UPDRS III
				*Flavonifractor*—positive correlation with UPDRS III
		*Bacillales—*negative correlation with UPDRS III		
		*Lactobacillales*	*Lactobacillaceae*	*Lactobacillus*—positive correlation with motor complications
			*Enterococcaceae*	*Enterococcus—*positive correlation with UPDRS III
		*Erysipelotrichales*	*Turicibacteraceae*	*Turicibacter*—positive correlation with UPDRS III
			*Erysipelotrichaceae*—positive correlation with UPDRS III	*Erysipelatoclostridium—*positive correlation with UPDRS III
		*Acidaminococcales*	*Acidaminococcaceae* positive correlation with UPDRS III	*Phascolarctobacterium*—positive correlation with UPDRS III
		*Selemonadales*	*Veillonellaceae*	*Megasphaera*—positive correlation with UPDRS III
	*Bacteroidota*	*Bacteroidales*	*Porphyromonadaceae*	*Parabacteroides*—positive correlation with UPDRS III
			*Bacteroidaceae*	*Bacteroides*—positive correlation with UPDRS III
			*Prevotellaceae*—negative correlation with UPDRS III	*Prevotella*—negative correlation with symptom severity
				*Paraprevotella*—negative correlation with UPDRS III/H&Y
	*Pseudomonadota*	*Enterobacteriales*	*Enterobacteriaceae*—positive correlation with UPDRS III	*Escherichia*—positive correlation with UPDRS III * species *E. coli*—positive correlation with UPDRS III
				*Shigella—*positive correlation with UPDRS III
				*Proteus*—positive correlation with UPDRS III
		*Pasteurellales*	*Pasteurellaceae*	*Haemophilus*—negative correlation with UPDRS III
		*Pseudomonadales*	*Pseudomonadaceae*	Pseudomonas* species *Pseudomonas_veronii*—negative correlation with UPDRS III
	*Verrucomicrobiota*	*Verrucomicrobiales*	*Akkermansiaceae*	*Akkermansia*—positive correlation with UPDRS III
	*Thermodesulfo-bacteriota*	*Desulfovibrionales*	*Desulfovibrionaceae*	*Desulfovibrio*—positive correlation with H&Y
				*Bilophila*—positive correlation with H&Y
	*Actinomycetota*	*Coriobacteriales*	*Coriobacteriaceae*	*Collinsella*—positive correlation with H&Y
		*Eggerthellales*	*Eggerthellaceae*	*Eggerthella*—positive correlation with H&Y
				*Adlercreutzia*—positive correlation with H&Y
	*Euryarchaeota*	*Methanobacteriales*	*Methanobacteriaceae*	*Methanobrevibacter* –positive correlation with H&Y
	*Lentisphaerota*	*Victivallales*	*Victivallaceae*	Victivalis* species *Victivalis_vividensis*—positive correlation with UPDRS III

*Species denotes a subcategory of genus which is species.

##### Phylum *Bacillota* (formerly *Firmicutes*)

Bacteria belonging to the *Bacillota* phylum have shown a mostly positive correlation with UPDRS III scores. For instance, the relative abundances of the genera *Peptococcus* and *Flavonifractor*, which belong to the *Clostridiales* order, have shown a positive correlation with UPDRS III scores (Baldini et al., 2020). This has also been shown in the case of the orders *Bacillales* and *Accidaminococcales*, more specifically its family *Acidaminococcacea*, as well as the genus *Phascolarctobacterium* (Li et al., 2019).

Regarding the *Eubacteriales* order, the relative abundance of the family *Lachnospiraceae* has been shown to have a negative correlation with UPDRS III (Barichella et al., 2019; Pietrucci et al., 2019). The relative abundance of the genus *Coprococcus* has shown a positive correlation with PD progression and symptom severity (Li et al., 2019; Zhang et al., 2020). The relative abundance of another member of this family, the genus *Tyzzerella*, has shown a positive correlation with UPDRS III (Li et al., 2019). Same has been shown with *Clostridium* cluster XIVa (Heintz-Buschart et al., 2018). The relative abundance of the genus *Blautia* has been shown, on the other hand, to have a negative correlation with motor complications in PD patients (Takahashi et al., 2022). Furthermore, genera from the *Oscillospiraceae* family have also shown a link to motor symptoms. In one study, the relative abundance of the genus *Anaerotruncus* has been shown to have a positive correlation with UPDRS III scores (Heintz-Buschart et al., 2018), while in the case of the genus *Faecalibacterium*, the relative abundance demonstrated a negative correlation with UPDRS III (Li et al., 2017) as well as with H&Y scales (Li et al., 2017; Weis et al., 2019). Similarly, in the case of the genus *Ruminococcus*, its relative abundance has also shown a negative correlation with UPDRS III scores (Li et al., 2017). In a different study, the relative abundance of the species *Ruminococcus_torques*, which is taxonomically counted as part of the *Mediterraneibacter* genus, has shown a positive correlation with UPDRS III (Li et al., 2019). Similarly, the relative abundance of the genus *Peptoniphilius*, which is a member of the *Peptoniphilaceae* family, has shown a positive correlation with H&Y scales (Weis et al., 2019).

Regarding the *Lactobacillales* order, the relative abundances of the family *Lactobaccilaceae* as well as the genus *Enterococcus* from the *Enterococcaceae* family, have shown a positive correlation with UPDRS III scores (Barichella et al., 2019). In a different study, the relative abundance of the genus *Lactobacillus* has shown a significant increase in patients with motor complications (Takahashi et al., 2022). Furthermore, the relative abundance of the genus *Turicibacter*, a genus belonging to the *Erysipelotrichia* class, has shown a positive correlation with UPDRS III scores (Cosma-Grigorov et al., 2020). Same has been shown in the case of the family *Erysipelotrichaceae* (Li et al., 2019) and its genus *Erysipelatoclostridium* (Rosario et al., 2021). In another study, the relative abundance of the genus *Megasphaera* has been shown to correlate positively with motor symptom severity, as demonstrated through H&Y scores (Li et al., 2017).

##### Phylum *Bacteroidota* (formerly *Bacteroidetes*)

In the *Bacteroidota* phylum the link between relative abundances and motor symptoms has been shown to be varied. A positive correlation with UPDRS III scores has been shown in families *Porphyromonadaceeae*, more specifically its genus *Parabacteroides* (Cosma-Grigorov et al., 2020; Zhang et al., 2020) and *Bacteroidaceae*, more specifically its genus *Bacteroides* (Lin et al., 2019). On the other hand, the relative abundance of the *Prevotellaceae* family has been shown to have a negative correlation with UPDRS III scores (Scheperjans et al., 2015; Aho et al., 2019). More specificially, a lower relative abundance of the genus *Prevotella* has been shown to be linked with earlier age of onset with a correlation in symptom severity (Aho et al., 2019). The abundance of the genus *Paraprevotella*, besides having a negative correlation with UPDRS III scores has also been shown to have a negative correlation with H&Y scales (Baldini et al., 2020).

##### Phylum *Pseudomonadota* (formerly *Proteobacteria*)

Like other bacteria in this review, members of the *Pseudomonadota* phylum have also shown a correlation to motor symptoms in PD. For instance, the relative abundance of the family *Enterobacteriaceae*, which is a part of the *Gammaproteobacteria* class, has shown a positive correlation both with motor symptoms and disease severity (Pietrucci et al., 2019). Moreover, the genera *Proteus, Escherichia*, and *Shigella*, which are a part of this family, have also individually shown a positive correlation between their relative abundances and UPDRS III (Li et al., 2017). Furthermore, the relative abundance of the species *E. coli*, a species belonging to the *Escherichia* genus, has been positively correlated with disease severity (Rosario et al., 2021). On the other hand, the relative abundance of the genus *Haemophilus*, which is a part of the *Pasteurellaceae* family, has shown a negative correlation with UPDRS scores (Li et al., 2017). This has also been shown with the species *Pseudomonas_veronii*, which belongs to the *Pseudomonaceae* family (Li et al., 2019).

##### Other

There have been studies that have reported representatives with correlation to PD from other phyli too. For instance, the relative abundance of the genus *Akkermansia*, from the phylum *Verrucomicrobiota*, has been shown to have positive correlation with UPDRS III scores (Heintz-Buschart et al., 2018). The relative abundance of the genus *Bilophila*, which is a part of the phylum *Thermodesulfobateriota*, has been shown to have a positive correlation with H&Y scales (Baldini et al., 2020) as well as disease progression and symptom severity (Zhang et al., 2020). The relative abundance of the *Desulfovibrio* genus has been shown to be positively correlated with higher scores on the H&Y scale (Murros et al., 2021). The relative abundance of genera *Colinsella, Eggerthella*, and *Adlercreutzia*, all a part of the *Actinomycetota* phylum, have also been shown to have a positive correlation with PD progression and symptom severity (Zhang et al., 2020), something which has also been demonstrated in the case of *Methanobrevibacter* genus, which is a part of the *Euryachaeota* phylum (Zhang et al., 2020; Rosario et al., 2021).

In the earlier mentioned study conducted by Rosario et al. (2021), the relative abundance of the species *Victivalis vadensis*, which belongs to the *Lentisphaerota* phylum, has also shown a positive correlation with UPDRS III scores in the same study.

#### Impact of microbiota on non-motor symptoms in Parkinson’s disease

Similarly to motor symptoms, research has shown a mostly positive correlation between the abundances of certain microbiota taxa and non-motor symptoms in PD (Table 4).

**TABLE 4:** Relative abundances of bacterial taxa in correlation with non-motor symptoms.

Kingdom	Phylum	Order	Family	Genus
*Bacteria*	*Bacillota*	*Eubacteriales*	*Oscillospiraceae*	*Ruminococcus—*positive correlation with NMSQ
				*Hydrogenoanaerobacterium*—negative correlation with MoCA
				*Oscillospira—*negative correlation with MoCA
				*Anaerotruncus*—positive correlation with depression
				*Mediterraneibacter* * species *Ruminococcus_torques*—positive correlation with NMSQ
			*Lachnospiraceae*	*Coprococccus—*positive correlation with NMSQ
				*Tyzzerella*—positive correlation with NMSQ
			*Clostridiaceae*	*Clostridium XIVb—*positive correlation with MMSE
				*Butyriciococcus*—positive correlation with MMSE
			*Christensenellaceae*—positive correlation with non-motor symptoms	
		*Acidaminococcales*	*Acidaminococcaceae*—positive correlation with NMSQ	*Acidaminococcus—*negative correlation with MMSE
		*Erysipelotrichales*	*Erysipelotrichaceae*—positive correlation with NMSQ	*Solobacterium*—negative correlation with MoCA
		*Lactobacillales*—negative correlation with NMSQ		
	*Bacteroidota*	*Bacteroidales*	*Odoribacteraceae*	*Odoribacter—*negative correlation with MMSE
				*Butyricimonas*—negative correlation with MMSE
			*Barnesiellaceae*	*Barnesiella—*negative correlation with MMSE
			*Rikenellaceae*	*Alistipes*—negative correlation with MoCA
			*Prevotellaceae—*negative correlation with IBS-like symptoms	*Prevotella*—negative correlation with IBS-like symptoms
	*Pseudomonadota*	*Burkholderiales*	*Oxalobacteraceae*	*Oxalobacter*—negative correlation with MoCA
			*Sutterellaceae*	*Sutterella*—negative correlation with MoCA
		*Enterobacteriales*	*Enterobacteriaceae*	*Escherichia—*positive association with GI dysfunction
	*Verrucomicrobiota*	*Verrucomicrobiales*	*Akkermansiaceae*	*Akkermansia*—positive correlation with NMSQ
	*Thermodesulfo-bacteriota*	*Desulfovibrionales*	*Desulfovibrionaceae*	*Desulfovibrio*—negative correlation with MMSE; linked to hyposmia
				*Bilophila*—negative correlation with MMSE
	*Actinomycetota*	*Bifidobacteriales*	*Bifidobacteriaceae*	*Bifidobacterium—*positive correlation with UPDRS I and constipation
	*Campylobacterota*	*Campylobacterales*	*Helicobacteraceae*	*Helicobacter*—negative correlation with MoCA
	*Synergistota*	*Synergistales*	*Synergistaceae*	*Pyramidobacter—*negative correlation with MoCA

*Species denotes a subcategory of genus which is species.

##### Phylum *Bacillota*

Besides having a connection to motor symptoms, bacteria from this phylum have been shown to have a link to MoCA and MMSE scores, as well as other non-motor symptoms.

For instance, the relative abundance of the class *Clostridia* has shown a negative correlation with MMSE (Li et al., 2022). This can also be seen at lower taxonomic levels, with the abundance of the genus *Hydrogenoanaerobacterium* showing a negative association with MoCA scores and the relative abundance of the genus *Ruminococcus* showing a negative association with MMSE (Ren et al., 2020).

In regards to the *Eubacteriales* order, the relative abundances of the genera *Coprococcus* and *Tyzzerella*, which are a part of the family *Lachnospiraceae*, have demonstrated a positive correlation with non-motor symptoms (Li et al., 2019). A higher relative abundance of the family *Christensenellaceae* has also been positively correlated with non-motor symptoms (Barichella et al., 2019). The relative abundance of the genus *Oscillospira*, which is a part of the *Oscillospiraceae* family, has shown a negative association with MoCA scores (Ren et al., 2020). Furthermore, changes in the relative abundance of the genus *Anaerotruncus* have been linked to depression (Heintz-Buschart et al., 2018). Moreover, the relative abundance of the earlier mentioned species *Ruminococcus_torques* has shown a positive correlation with NMSQ (Li et al., 2019). On the other hand, the relative abundances of *Clostridium XIVb* and the genus *Butyriciococcus*, which are a part of the family *Clostridiaceae*, have shown a positive association with MMSE (Qian et al., 2018).

The *Accidaminococcales* order also showed a link to non-motor symptoms, with the relative abundance of the family *Acidaminococcaceae*, demonstrating a positive correlation with NMSQ scores (Li et al., 2019). In a different study, the relative abundance of its genus *Acidaminococcus* was negatively associated with MMSE scores (Ren et al., 2020).

Regarding the *Erysipelotrichiales* order, the relative abundance of the genus *Solobacterium*, which is a part of the family *Erysipelotrichidae*, has shown a negative association with MoCA scores (Ren et al., 2020). In the study conducted by Li et al. (2019), the family *Erysipelotrichaceae* has shown a similar connection to non-motor symptoms, with its relative abundance positively correlating with NMSQ scores. On the other hand, in the same study, the relative abundance of the order *Lactobacillales* was negatively correlated with NMSQ (Li et al., 2019).

##### Phyli *Bacteroidota* and *Pseudomonadota*

A study conducted by Ren et al. (2020) reveals several genera from both phyla which have been shown to have a negative correlation between their relative abundance and MMSE/MoCA. In the phylum *Bacteroidota*, this has been shown with the family *Odoribacteraceae*. The relative abundances of the genera *Odoribacter* and *Butyricimonas* have been negatively correlated with MMSE. Same has been shown for the genus *Barnesiella*, which is a member of the *Barnesiellaceae* family. Furthermore, the relative abundance of genus *Alistipes*, a part of the *Rikenellaceae* family, has shown a negative association with MoCA scores. The same connection was established for members of the phylum *Pseudomonadota*. In the *Betaproteobacteria* class, the relative abundance of the genus *Oxalobacter* has shown a negative association with MMSE, while the abundance of the genus *Sutterella* has shown a negative association with MoCA (Ren et al., 2020).

The link to GI dysfunction has also been explored. In one study, representatives of the *Bacteroidota* phylum, more specifically the family *Prevotellaceae* as well as its genus *Prevotella*, have shown a lowered abundance in PD patients with irritable bowel syndrome-like symptoms (Mertsalmi et al., 2017). In a different study, the relative abundance of the species *E. coli*, which belongs to *Gamaproteobacteria*, has shown a positive correlation with gastrointestinal (GI) dysfunction (Rosario et al., 2021). In the same study, the earlier mentioned *Victivallis vadensis* has shown a similar connection.

##### Other

There have been additional genera reported to influence non-motor symptoms in PD. In one study, the relative abundance of the earlier mentioned *Akkermansia*, besides being connected to motor symptoms, demonstrated a positive correlation between its relative abundance and NMSQ (Li et al., 2019). The relative abundances of *Bilophila* and *Desulfovibrio*, two genera from the family of *Desulfovibrionaceae*, which is a part of the *Thermodesulfobacteriota* phylum, have been found to have a negative correlation between relative abundance and MMSE (Ren et al., 2020). Also, the genus *Desulfovibrio* has been found to be more abundant in patients with hyposmia (Murros et al., 2021).

In one study, the relative abundance of the genus *Bifidobacterium*, which is a part of the *Actinomycetota* phylum, has been associated with constipation (Baldini et al., 2020) and its relative abundance has also been found to have a positive correlation with UPDRS I scores, mainly through its link with hallucinations (Minato et al., 2017).

In the earlier mentioned study by Ren et al. (2020), two more genera have been linked to lower performances on MoCA and MMSE scores. For instance, the relative abundance of the genus *Helicobacter*, which belongs to the *Campylobacterota* phylum, has shown a negative association with MoCA scores, while on the other hand, the relative abundance of the genus *Pyramidobacter*, which is a part of the *Synergistotta* phylum, has shown a negative association with MMSE (Ren et al., 2020).

## Discussion

In the last decade, more studies have tackled the effect of gut microbiome alteration on the emergence and development of neurodegenerative diseases, with PD being an especially interesting target for research due to the wide range of different motor and non-motor symptoms.

The studies collected in this systematic review have mostly correlated the relative abundance of various gut microbiota taxa with UPDRS III scores and H&Y scale, used to express motor symptom severity and disease severity, while the non-motor symptoms were tested mainly through MoCA, MMSE, and NMSS. Oral and nasal microbiota was not considered for this review, due to a limited number of research. Multiple confounders such as diet, therapy and comorbidities were partially considered in the research conducted by the authors. For instance, in a study by Li et al. (2019), several correlations were found between certain microbiota taxa and clinical scales when analyzed both on the PD patients and the healthy controls together. However, after analyzing them individually, no significant correlations were found in either the PD group or the controls (Li et al., 2019). This difference could reflect the impact of the disease as a general state, rather than present as a connection between specific microbiota and individual symptoms and clinical scales showing that the confounding factors should be carefully considered when conducting microbiome research regardless of a potential link between the relative abundance of specific microbiota taxa and s clinical scales that might present itself in the initial results. It should also be noted that the studies in this review have demonstrated a variability in the number of participants, with numbers ranging from 20 to 350, with some research not including healthy controls.

Another problem could arise in the varied methodology used for the analysis of microbiota composition (Table 2). While most of the studies used the V3–V4 regions of 16S RNA for amplification and sequencing (Qian et al., 2018; Aho et al., 2019; Barichella et al., 2019; Pietrucci et al., 2019; Baldini et al., 2020; Cosma-Grigorov et al., 2020; Ren et al., 2020; Takahashi et al., 2022) some of the studies used other regions such as V1–V3 regions (Scheperjans et al., 2015; Mertsalmi et al., 2017), purely V4 regions of 16S RNA (Heintz-Buschart et al., 2018; Li et al., 2019; Lin et al., 2019; Zhang et al., 2020), V3–V5 (Li et al., 2017), V4–V5 regions (Weis et al., 2019; Li et al., 2022). A study by Heintz-Buschart et al. (2018), besides using 16S RNA also used the 18S RNA. In a study conducted by Minato et al. (2017), besides analyzing the 16S RNA, the 23S RNA was also included, and a special protocol was used, called SYBR Green 1, with a selected number of only 19 bacterial taxa used. A study by Rosario et al. (2021) used previously acquired metagenomic data from a German PD Cohort conducted earlier and did not specify the methods of amplification and sequencing. In a different study, specific primers for the 16S rRNA were used for detection of only one genus and its subspecies, the genus being *Desulfovibrio* (Murros et al., 2021). It is apparent that through the inclusion of, not only different regions of 16S RNA, but also different RNA-s altogether, the results could significantly vary. The methods of OTU (Operational Taxonomic Unit) designation were also varied, including Mothur, QIIME pipeline, QIIME2 pipeline, USEARCH, UPARSE, and databases such as GreenGenes, SILVA, among others (Table 2). The biggest concern, however, is the extremely variable microbiota data and statistical analysis methodology, which in turn could lead to potentially spurious correlations between clinical scales and relative abundances acquired and analyzed through these various methods. Due to microbiome data being compositional in nature, compositionality-aware methods for correlation and differential abundance should be used, such as SparCC and Spearman’s rank correlation coefficient, when it comes to correlation and ALDEx2/ANCOM for differential abundance (Gloor et al., 2017). A number of studies represented in this review did in fact use some form of compositional analysis such as Spearman’s rank correlation coefficient or SparCC to calculate and present the correlations between relative abundances of gut microbiota and symptoms of PD (Table 2). However, methods like ANCOM were used to a lesser extent (Heintz-Buschart et al., 2018; Aho et al., 2019; Lin et al., 2019). A model for a compositional approach as opposed to a standard one was proposed in a paper by Gloor et al. (2017), and could help in developing a standardized approach for microbiota analysis in the future.

With all of this in mind, the results of this review and the studies encompassed should be interpreted cautiously.

When it comes to motor symptoms, the relative abundance of gut microbiota taxa has been often shown to positively correlate with UPDRS III scores and H&Y scale. The exception to this was microbiota belonging to the order *Bacillales* (Li et al., 2019), families *Prevotellaceae* (Scheperjans et al., 2015; Aho et al., 2019), and *Lachnospiraceae* (Barichella et al., 2019; Pietrucci et al., 2019), while on a genus level, this has been shown with genera *Ruminococcus*, *Haemophilus* (Li et al., 2017), *Prevotella* (Aho et al., 2019), *Flavonifractor, Paraprevotella* (Baldini et al., 2020), *Blautia* (Takahashi et al., 2022), *Faecalibacterium* (Li et al., 2017; Weis et al., 2019) and *Prevotella* (Aho et al., 2019), and species *Pseudomonas_veronii* (Li et al., 2019). It can be thus hypothesized that the increase in relative abundance of various microbiota taxa could lead to more expressed motor symptoms in PD patients. It should be noted, however, that the results focused on higher taxonomic instances should be interpreted more carefully, especially with regards to the microbiota families and classes, since sometimes there are members of said groups that have an entirely opposite correlation when observed on a genus level. This is the case of *Coprococcus* (Li et al., 2019; Zhang et al., 2020), which has been shown to have a positive correlation with UPDRS III scores and motor symptom severity, in contrast to *Lachnospiraceae* family of which it is a part of. Another factor is the correlation between relative abundances of different microbiota taxa in the same sample, whereas the increase of relative abundances on certain taxonomic levels, such in the case of *Ruminococcaceae*, has been shown to compensate the lower levels of *Prevotellaceae* (Scheperjans et al., 2015).

Considering non-motor symptoms, the microbiota taxa identified in this review has been found to have a mostly negative correlation with non-motor symptoms, apart from the order *Lactobacillales* which showed a negative correlation with NMSQ scores (Li et al., 2019) as well as the genera *Clostridium XIVb* and *Butyriciococcus* (Qian et al., 2018) which demonstrated positive correlations to MoCA/MMSE scores. The link between microbiota and cognitive decline is being researched regarding Alzheimer’s disease (Khedr et al., 2022) and the etiopathogenesis behind changes leading to cognitive deterioration potentially modulated by microbiome alterations are yet to be discovered in both diseases, with the possibility of shared mechanisms. It should also be noted that direct correlation between microbiota and non-motor symptoms is still under question, since both cognitive decline (Fang et al., 2020) and changes in microbiome (Li et al., 2017; Weis et al., 2019; Cosma-Grigorov et al., 2020; Murros et al., 2021; Rosario et al., 2021) have been shown as an intrinsic part of later disease stages. A negative connection to specific symptoms has been found in the case of the species *E. coli*, which is linked to GI dysfunction in PD patients (Rosario et al., 2021) and the genus *Desulfovibrio*, which has been linked to hyposmia (Murros et al., 2021). GI dysfunction is a staple of PD (Lubomski et al., 2020), but it poses the question of whether the microbiome changes are behind GI dysfunction or a cause for it. The link to hyposmia, which is thought to be caused by the early deposition of Lewy pathology in the olfactory bulb (Fullard et al., 2017), and microbiota could play a part in this through the earlier mentioned gut-brain hypothesis.

The mechanistic answer for these changes could lie in the metabolites of microbiota. One of the most prominently researched ones are the short-chain fatty acids (SCFA). In one study, the relative abundances of the species *Ruminococcus* sp. *AM07 15* and *Clostridiales bacterium NK3B98* have shown a correlation with the plasma and fecal levels of SCFA, most notably propionic acid. Furthermore, the same study showed that the decreased fecal levels and increased plasma levels of SCFA, most notably propionic acid, had a positive correlation to UPDRS III scores (Chen et al., 2022). This has also been shown in a different case-control study where serum level of propionic acid was correlated with UPDRS III scores, MMSE and Hamilton Depression Scale (HAM-D) (Wu et al., 2022). In a different study conducted by Aho et al. (2019), the genus *Prevotella* has been linked with a higher butyric acid production, which has been shown to postpone the age of disease onset in PD patients. The species *Akkermansia municiphila*, which belongs to the *Verrucomicrobiota* phylum, has shown a role in taurine metabolism, mainly through lowering plasma taurine levels, which in turn has a negative effect on UPDRS III scores. Same has been shown in the case of *Bilophila wadsworthia*, part of the *Thermodesulfobacteriota* phylum (Hertel et al., 2019). Another member of this phylum, the genus *Desulfovibriobacteria* has been proposed to produce magnetite as well as hydrogen sulfite which could accelerate alpha-synuclein aggregation (Murros et al., 2021).

In general, the limitation of the field is a scarce quantity of studies connecting microbiota abundance and metabolism with PD symptoms, and more research is needed to confirm the causal link between the two. When looking at the study design, clearly there is a lack of randomized controlled studies, case control studies with *de novo* patients and longitudinal studies. These are required to confirm the correlations mentioned in this systematic review, but also to highlight whether these changes are intrinsic to the disease or are perhaps a consequence of therapy as well. We are currently conducting a longitudinal study with *de novo* patients (Clinicaltrials.gov, NCT05008094) and are looking to add to the current knowledge of both symptom and abundance correlation, and the effects of therapy on the composition of microbiota.

All of this is important when looking at potential future therapeutic options, since gut microbiota can be altered by various intrinsic and extrinsic factors, which could in turn potentially influence the severity of symptoms of PD and other neurodegenerative diseases. For instance, antibiotics have been shown a potential benefit in a study conducted by Pu et al. (2019), where an antibiotic cocktail (ampicillin, neomycin sulfate, metronidazole) was applied in a MPTP rodent model. This caused changes in microbiome composition compared to the control group and countered the neurotoxic effects of MPTP (Pu et al., 2019). In a different study, the antibiotic rifampicin has been shown to inhibit α-synuclein fibrillation, a pathological mechanism behind PD (Li et al., 2004). Probiotics have also been explored. In one study, probiotic mixtures of different bacteria have been shown in one study to reduce dopaminergic neuron loss as well as increase dopamine levels (Srivastav et al., 2019). The Mediterranean diet has shown a beneficial effect in Alzheimer’s disease, but also in PD. The diet has been shown to be rich in *Lactobacilli*, and the adherence to the diet lowered the odds for both Alzheimer’s disease and PD (Alcalay et al., 2012). Another interesting study researched the potential for enema application as a modulation of microbiota composition. The UPDRS III scores improved after enema, with lowered abundances of the family *Ruminococcaceae* and the genus *Clostridium* (Hegelmaier et al., 2020). Another method that has been explored is FMT. In one study, colonic FMT has been shown to decrease UPDRS III, NMSQ, PDQ-39, HAM-D, and Hamilton Anxiety Scale (HAM-A) scores in a small group of 10 PD patients with only a minor number of mild self-limiting side effects, mostly pertaining to the GI tract (Xue et al., 2020). It is apparent that therapeutic intervention on the microbiome level, be it on a medical or a dietary, could increase the overall therapeutic yield and response to medication, or even potentially postpone the initial symptoms of the disease, something which should be further explored.

## Conclusion

It is apparent that the link between the microbiome and the neurodegeneration that could lead to motor and non-motor symptoms of PD complex and multifactorial. The relative abundance of specific microbiota taxa has been consistently shown to be correlated with symptom severity, either positively or negatively, but the causal link is still in question. The mechanistic answer could lie in the products of microbiota metabolism, which have also been linked to symptom severity through intricate metabolic pathways that are under influence of various confounding factors, with PD being just one part of the bigger picture. In a clinical setting, therapeutic interventions have already been explored regarding microbiome manipulation, showing promising results, be it through the use of antibiotics, probiotics, diet changes, or more specific methods such as enema application and FMT. Combined with established PD treatment, these methods could enhance the overall therapeutic success and provide a more personalized approach to each patient. Further research is thus warranted in the field, with a focus on both abundance and metabolic function of microbiota in relation to motor and non–motor symptoms, along with studies greater in quality, such as randomized controlled studies and case control studies with *de novo* patients and longitudinal studies, as well as more standardized methods for isolation and compositional data analysis.

## Author contributions

EP and VV conceptualized the systematic review. EP, VR, MH, ZT, NS-Č, AK, MK, GH, BP, and VV developed and consulted on the search strategy and methodology. EP, VR, MH, and AK assisted with screening article. EP, VR, and MH abstracted data from the article. EP drafted the manuscript. All authors reviewed, edited, assisted with writing subsequent drafts of the manuscript, and approved the final version of the manuscript.
